# Effect of dipeptidyl peptidase-4 inhibitors on tumor necrosis factor alpha levels in patients with type 2 diabetes mellitus

**DOI:** 10.1186/s40001-024-01955-9

**Published:** 2024-07-12

**Authors:** Lijia Zhao, Jie Meng, Xueyan Bai, Donglei Zhang, Xingsheng Yang, Yu Yang, Gaojun Cai, Xin Liu

**Affiliations:** 1 Department of Cardiology, Beijing Tiantan Hospital, Capital Medical University, Beijing, China; 2 Department of Pathology, Beijing TongRen Hospital, Capital Medical University, Beijing, China; 3 Department of Hemotology, Beijing Tiantan Hospital, Capital Medical University, Beijing, China; 4 Department of Hematology, Zhongnan Hospital, Wuhan University, Wuhan, China; 5 Department of Cardiology, Wujin Hospital, Jiangsu University, Changzhou, Jiangsu China; 6 Department of Pharmacy, Beijing Tiantan Hospital, Capital Medical University, Beijing, China

**Keywords:** Dipeptidyl peptidase-4 inhibitors, TNF-alpha, Type 2 diabetes mellitus, Randomized controlled trials

## Abstract

**Aims:**

Dipeptidyl peptidase-4 inhibitors (DPP-4i) served as oral antidiabetic agents for treatment of type 2 diabetes mellitus (T2DM). Although an action on glucose homeostasis was identified, no well-rounded illustration had been established on the changes of tumor necrosis factor alpha (TNF-alpha) levels during DPP-4i treatment. This study aimed to explore the anti-inflammatory effect of DPP-4i on TNF-alpha in patients with T2DM.

**Methods:**

PubMed, Embase and Cochrane Library were systematically searched from inception to May 31, 2024. Randomized controlled trials exploring the impact of DPP-4i on TNF-alpha levels were identified. Risk of bias was assessed according to the Cochrane criteria. A fixed or random-effects model was selected to pool estimate on whether the heterogeneity was present. Subgroup analysis were performed to explore the potential factors that influenced heterogeneity. Related meta-analysis was conducted with the software of Revman 5.3 and STATA 12.0.

**Results:**

Eleven trials involving 884 participants with T2DM were included. Pooled estimates suggested that DPP-4i did not significantly modulate TNF-alpha levels (WMD, − 0.70, 95% CI − 1.94 to 0.53, *P* = 0.26) in T2DM. DPP-4i produced a significant effect on TNF-alpha (WMD, − 4.50 pg/mL, 95% CI − 4.68 to − 4.32, *P* < 0.00001) when compared to placebo, and a comparable effect was demonstrated on TNF-alpha (WMD, 0.10 pg/mL, 95% CI − 0.11 to 0.30, *P* = 0.35) in comparison with active agents. Estimate was stable according to the sensitivity test. Subgroup analysis revealed that heterogeneity might not correlate with baseline glycated hemoglobin (HbA1c), age or treatment duration.

**Conclusions:**

A significant effect of DPP-4i on TNF-alpha levels was present in T2DM when compared to placebo. Administration of DPP-4i produced no significant effect on TNF-alpha in comparison with active comparators. Further studies with large samples should be performed to illustrate the impact of DPP-4i on TNF-alpha levels in T2DM.

*Trial registration* International Prospective Register for Systematic Review (PROSPERO) number: CRD42020185479

**Supplementary Information:**

The online version contains supplementary material available at 10.1186/s40001-024-01955-9.

## Background

The prevalence of diabetes had reached 8.5% across the world in 2014, and 90% patients were diagnosed with type 2 diabetes mellitus (T2DM). An increased incidence of morbidity or mortality could be attributed to the onset and development of macro- or microvascular complications in T2DM [1]. Epidemiological evidences had established a close link between inflammation and diabetic complications. It had been reported that tumor necrosis factor alpha (TNF-alpha) played a key role in activating inflammatory response, thus presenting a proinflammatory phenotype in patients with T2DM [2]. In addition, an increased release of TNF-alpha was commonly induced in hyperglycemia setting [3]. TNF-alpha interacted with specific receptors and activated inflammatory signaling pathway, contributing to the development of diabetic complications [4].

Traditional antidiabetic agents mainly focused on modulating glucose levels before 2006 [5]. Although sulfonylureas and thiazolidinediones effectively reduced glucose levels, these regimens produced no favorable effect on the prognosis of diabetes or related complications [6]. In the past two decades, novel antidiabetic agents were developed to improve glucose homeostasis with unique mechanisms [7]. Among these multiple classes, dipeptidyl peptidase-4 inhibitors (DPP-4i) served as the agent of incretin mimetics. DPP-4i effectively increased glucagon-like peptide-1 (GLP-1) concentrations via interacting with the protease of DPP-4. DPP-4i were firstly introduced for treatment of patients with T2DM in 2006. These agents effectively improved glucose homeostasis without increasing the risk of hypoglycemia or weight gain [8]. In addition, DPP-4i produced an improving effect on the function of pancreatic β-cell in fasting and postprandial states [9]. Despite a clear effect on glucose balance, no well-rounded association of DPP-4i with TNF-alpha levels had been illustrated in T2DM. This study aimed to help demonstrate the impact of DPP-4i on modulating TNF-alpha levels in T2DM.

## Methods

### Search strategy

This study was performed according to Preferred Reporting Items for Systematic reviews and meta-analysis (PRISMA) statement [10]. Multiple databases including PubMed, Embase and Cochrane Library were searched for randomized controlled trials (RCTs) from inception to May 31, 2024. The inclusion criteria were recorded as follows: (i) an effect of DPP-4i therapy on TNF-alpha was studied; (ii) relative information on the baseline and post-treatment renal parameter was recorded or a change of TNF-alpha was indicated; and (iii) patients were diagnosed with T2DM. The intervention group was set as DPP-4i treatment, while comparators were set as placebo or other antidiabetic agents except for DPP-4i. Additional trials were identified with a hand-searched method from the reference lists. Related first or corresponding authors were contacted when TNF-alpha value was missing. Searching items were used to identify eligible studies: (“dipeptidyl peptidase-4 inhibitors” OR “DPP-4i” OR “DPP-4 inhibitors”) AND (“type 2 diabetes” OR “type 2 diabetes mellitus” OR “T2DM”) AND (“randomized controlled trials” OR “RCT”). The exclusion criteria were as follows: (i) non-human studies; (ii) lack of detailed information on TNF-alpha; (iii) non-RCTs or narrative reviews; and (iv) non-diabetic patients or non-DPP-4i intervention.

### Quality evaluation

Quality evaluation of RCTs was performed according to the Cochrane Handbook by two researchers independently [10]. Seven items were evaluated with random sequence generation, allocation concealment, blinding of participants and personnel, blinding of outcome assessment, incomplete outcome data, selective outcome reporting and other potential sources of bias. A low risk of bias was marked as a judgement of ‘yes’, while a high risk of bias was given a mark of ‘no’. The item of ‘unclear’ represented as the unclear risk of bias. Two researchers discussed or consult with a third reviewer if discrepancies were present among these studies.

### Certainty of evidence

The quality of evidence for TNF-alpha in the identified RCTs was evaluated with the GRADE methodology and GRADE Pro tool [11]. Four categories of the quality of the evidence were offered by the GRADE system as follows:

- High: high confidence in the match between the actual and estimated effect;
- Moderate: moderate confidence in the effect estimate. There is a possibility that the actual effect is far from the estimated effect;
- Low: limited confidence in the estimate of the effect. The actual effect may be far from the estimated effect;
- Very low: low confidence in the estimated effect. The actual effect is very likely to be different from the estimated effect.

### Data extraction

Two researchers independently extracted information into a standardized form. Detailed information was recorded including first author, publication year, country of origin, DPP-4i or control intervention, sample size, number of male patients, body mass index (BMI), mean age, diabetes duration, treatment duration and TNF-alpha concentrations at baseline. The longest group was extracted when multiple follow-ups were recorded in the same study. The primary outcome was set as a mean change of TNF-alpha concentration.

### Data analysis

Meta-analysis was performed with Revman 5.3 and STATA 12.0 (software). The effect size of TNF-alpha was analyzed with weighted mean difference (WMD) and 95% confidence interval (CI). A fixed-effects or random-effects model was selected based on the potential heterogeneity, which was evaluated with a Chi-square test and quantified with *I*^2^ index. The index of *I*^2^ ranging from 0 to 50% indicated no significant heterogeneity. In contrast, a marked heterogeneity was produced when *I*^2^ was greater than 50%. Sensitivity test was performed by using the leave-one-out method. Publication bias was examined with Begg’s and Egger’s tests when there were at least five studies in the pooled analysis. Furthermore, subgroup analysis was performed to explore potential factors that potentially changed heterogeneity based on glycated hemoglobin (HbA1c) at baseline, age and treatment duration.

## Results

### Data extraction

**Fig. 1:**
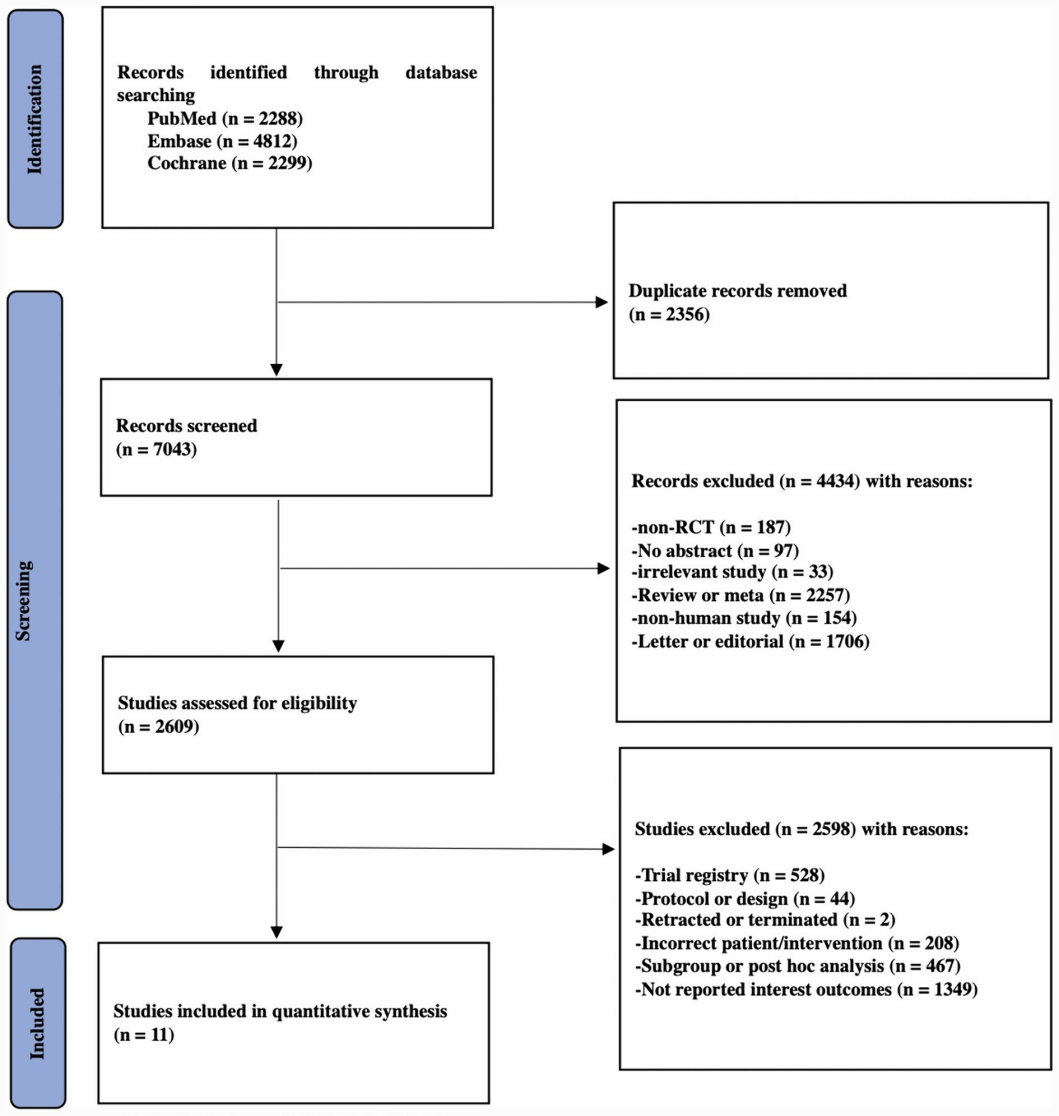
PRISMA flowchart for study selection

A total of 9399 records from these three databases after performing a systematic search. According to the established inclusion and exclusion criteria, 11 RCTs involving 884 participants were included (Fig. 1). Sitagliptin and vildagliptin were mostly chosen for the treatment of T2DM, and the remaining patients were administered with alogliptin and anagliptin, respectively. The related searching strategy was based on the inclusion criteria (supplementary file 1). The PRISMA Checklist was also presented in detail (supplementary file 2).

### Characteristics of included studies

The characteristics of each RCT were demonstrated and related trials were published between 2010 and 2020 (Table 1). Participants with a mean age range of 54 to 65 year were recruited, and the related parameter of BMI ranged from 24 to 29 kg/m^2^. Except for two studies with multicenter design, the other studies were single-center, and the other five studies were conducted in Japan. Two studies were set as placebo control, and nine studies were marked as active comparators. The longest follow-up was 12 months, while the shortest time was 3 months. In the largest sample study, 137 patients were included. However, a sample size of 24 was recruited in the smallest study. Among these participants, 472 patients were administered with DPP-4i monotherapy, or added on the basis of metformin or other antidiabetic agents. In addition, 222 patients were treated with sitagliptin, 196 with vildagliptin, 42 with alogliptin and 12 with anagliptin. In the control group, 412 participants were treated with placebo (*n* = 69) or other antidiabetic agents (*n* = 343).

**Table 1:** General characteristics of the studies included

Study, year	Location	Treatment arm (*n*)	HbA1c (%)	Male (*n*)	Age (years)	BMI (kg/m^2^)	Diabetes duration (years)	Treatment duration (months)	TNF-alpha (pg/mL)
Asahara, 2013 [37]	Japan	sita:24	8.3 ± 0.2	11	62.0 ± 2.3	25.5 ± 0.9	NS	3	10.2 ± 1.0
		con:24	8.2 ± 0.2	14	58.0 ± 2.4	26.5 ± 0.7			8.8 ± 0.8
Tian, 2016 [38]	China	sita:88	8.1 ± 0.6	44	54.0 ± 8.1	25.9 ± 4.1	Newly diagnosed	3	12.0 ± 0.5
		pla:45	8.2 ± 0.5	22	54.0 ± 8.1	25.5 ± 3.1			11.7 ± 0.3
Derosa, 2010 [39]	Italy	sita:75	8.5 ± 0.9	37	57.0 ± 5.0	27.9 ± 1.5	5.0 ± 2.0	12	3.8 ± 1.1
		met:76	8.4 ± 0.8	39	58.0 ± 6.0	27.7 ± 1.3	6.0 ± 3.0		4.0 ± 1.4
Kitao, 2017 [40]	Multicenter	vil:48	7.3 ± 0.6	26	62.0 ± 14.3	25.7 ± 4.1	5.4 ± 3.5	3	1.1 ± 0.5
		met:48	7.2 ± 0.8	31	60.0 ± 18.0	26.1 ± 4.7	6.5 ± 3.5		1.2 ± 0.4
Nomoto, 2016 [41]	Multicenter	sita:48	7.4 ± 0.6	33	62.0 ± 15.0	25.7 ± 3.9	NS	6.5	NS
		glim:55	7.4 ± 0.4	29	60.0 ± 8.0	25.2 ± 3.5			
Phrommintikul, 2019 [42]	Thailand	vil:24	8.2 ± 1.2	12	63.9 ± 7.6	24.9 ± 3.2	> 5	6	0.1 ± 0.2
		dapa:25	8.1 ± 1.4	11	62.6 ± 8.3	25.6 ± 3.0			0.1 ± 0.2
Takeshita, 2015 [43]	Japan	sita:30	6.6 ± 0.5	15	63.5 ± 12.0	24.6 ± 3.8	9.7 ± 9.2	4	2.9 ± 1.7
		miti:30	7.0 ± 0.8	15		24.2 ± 4.6			2.1 ± 1.9
Takeshita, 2015 [44]	Japan	vil:58	8.1 ± 1.2	40	64.5 ± 12.7	24.5 ± 4.6	NS	3	1.5 ± 1.0
		lira:54	8.0 ± 0.9	39	64.9 ± 1.9	25.4 ± 4.8			1.2 ± 0.5
Dei Cas, 2017 [45]	Italy	vil:40	7.7 ± 0.2	26	61.0 ± 9.0	29.1 ± 2.0	7.0 ± 2.3	12	1.4 ± 0.4
		glib:24	7.7 ± 0.2	17	63.0 ± 10.0	28.9 ± 2.9	5.0 ± 3.0		1.6 ± 0.4
Takeshita, 2019 [46]	Japan	alo:42	7.5 ± 1.0	29	63.8 ± 10.5	25.4 ± 6.1	10.2 ± 10.4	3	1.6 ± 1.5
		met:42	7.4 ± 1.2	29	63.1 ± 13.1	24.4 ± 4.0	14.1 ± 13.0		2.4 ± 4.2
Onoue, 2020 [47]	Japan	anag:12	7.1 ± 0.5	7	64.8 ± 12.4	26.9 ± 6.0	7.7 ± 4.6	6	1.3 ± 0.4
		con:12	7.3 ± 0.5	8	62.9 ± 14.7	25.6 ± 3.5	7.4 ± 8.3		1.1 ± 0.4

Values are expressed as the mean ± SD

*n* number of participants per group, *HbA1c* glycated hemoglobin, *sita* sitagliptin, *vild* vildagliptin, *alo* alogliptin, *anag* anagliptin, *met* metformin, *pla* placebo, *dapa* dapagliflozin, *miti* mitiglinide, *con* conventional treatment, *lira* liraglutide, *piog* pioglitazone, *vog* voglibose, *glim* glimepiride, *lin* linagliptin, *glib* glibenclamide, *insu* insulin, *cos* chitosan oligosaccharide, *NS* not stated

### Quality evaluation

**Fig. 2:**
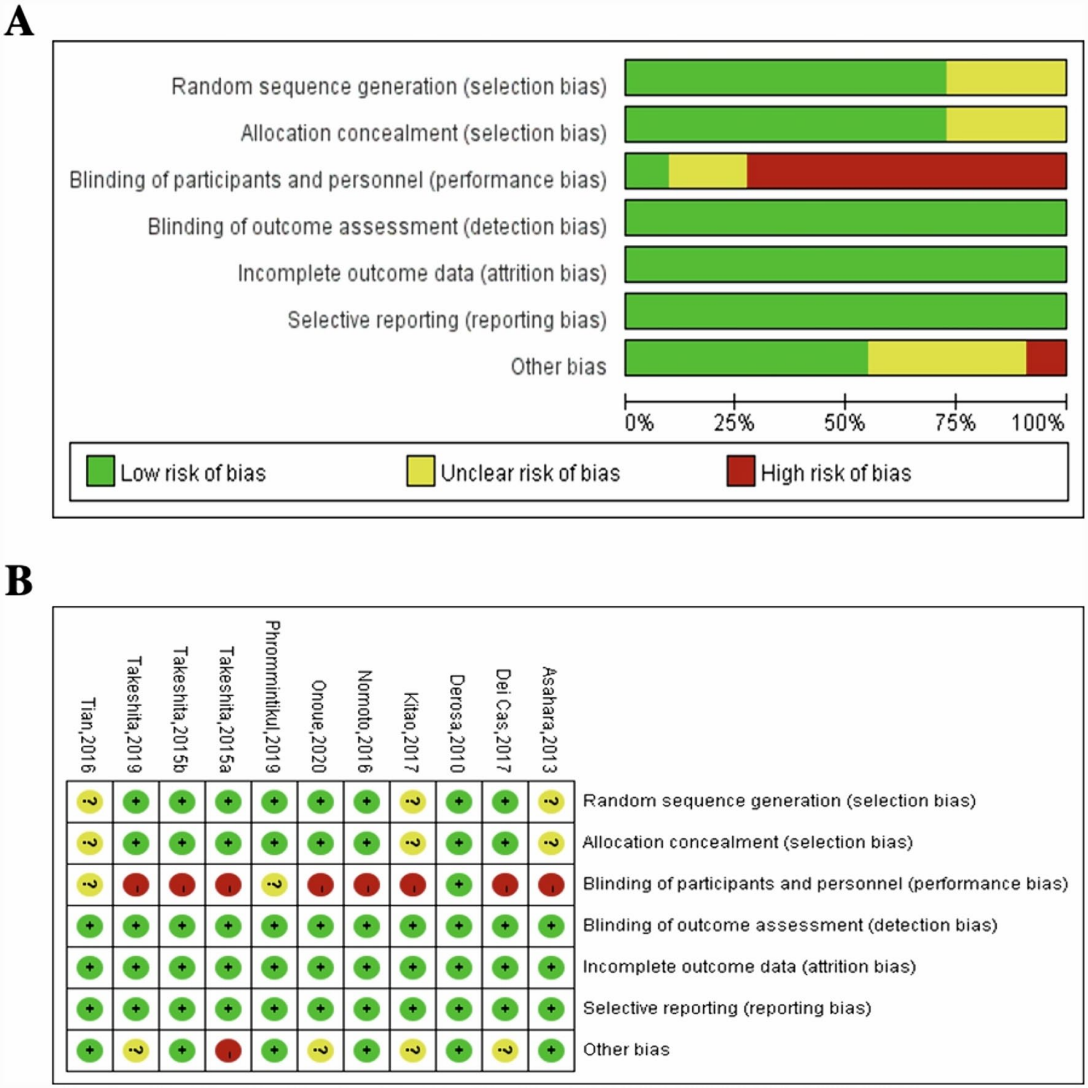
Assessment of bias in the included studies according to the Cochrane criteria

Quality evaluation of RCTs was performed according to the Cochrane criteria (Fig. 2). The risk of bias was mainly marked as “unclear” in three settings of random sequence generation and allocation concealment. All studies performed the procedure of blinding of outcome assessment, whereas eight studies had performance bias due to a lack of implementation of blinding methods.

### Certainty of evidence

The GRADE assessment for TNF-alpha related to DPP-4i is demonstrated in Table 2. According to the GRADE system, the quality of evidence for RCTs assessing the effect of DPP-4i for TNF-alpha was rated as very low.

**Table 2:** GRADE assessment for TNF-alpha changes during DPP-4i treatment in T2DM

DPP-4i compared to control group for [health problem]					
Patient or population: patients with [health problem]					
Settings: intervention: DPP-4i; comparison: control group					

Outcomes	Illustrative comparative risks* (95% CI)		Relative effect (95% CI)	No. of participants (studies)	Quality of the evidence (GRADE)	Comments
	Assumed risk	Corresponding risk				
	Control group	DPP-4i				
TNF-alpha		The mean TNF-alpha in the intervention groups was 0.70 lower (1.94 lower to 0.53 higher)		884 (11 studies)	${\oplus} {\ominus} {\ominus} {\ominus}$ very low^a,b,c^	

* The basis for the assumed risk (e.g., the median control group risk across studies) is provided in footnotes. The corresponding risk (and its 95% confidence interval) is based on the assumed risk in the comparison group and the relative effect of the intervention (and its 95% CI); a, Large heterogeneity I^2^ = 100%; b, 95% CI including invalid data; c, Funnel plot asymmetry

### Effect of DPP-4i on TNF-alpha

**Fig. 3:**
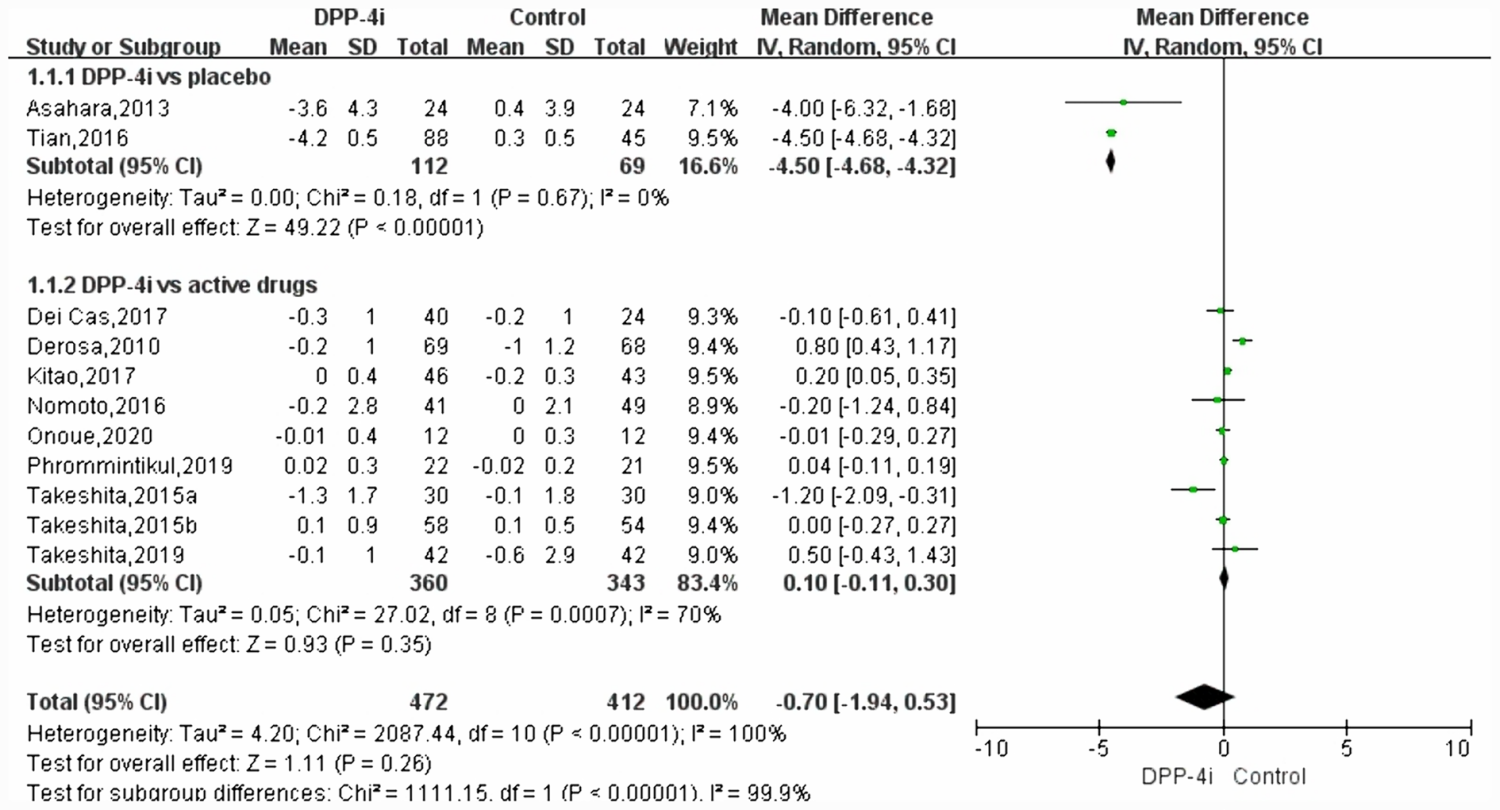
Forest plot for the analysis of the effect of DDP-4i on TNF-alpha concentrations

**Fig. 4:**
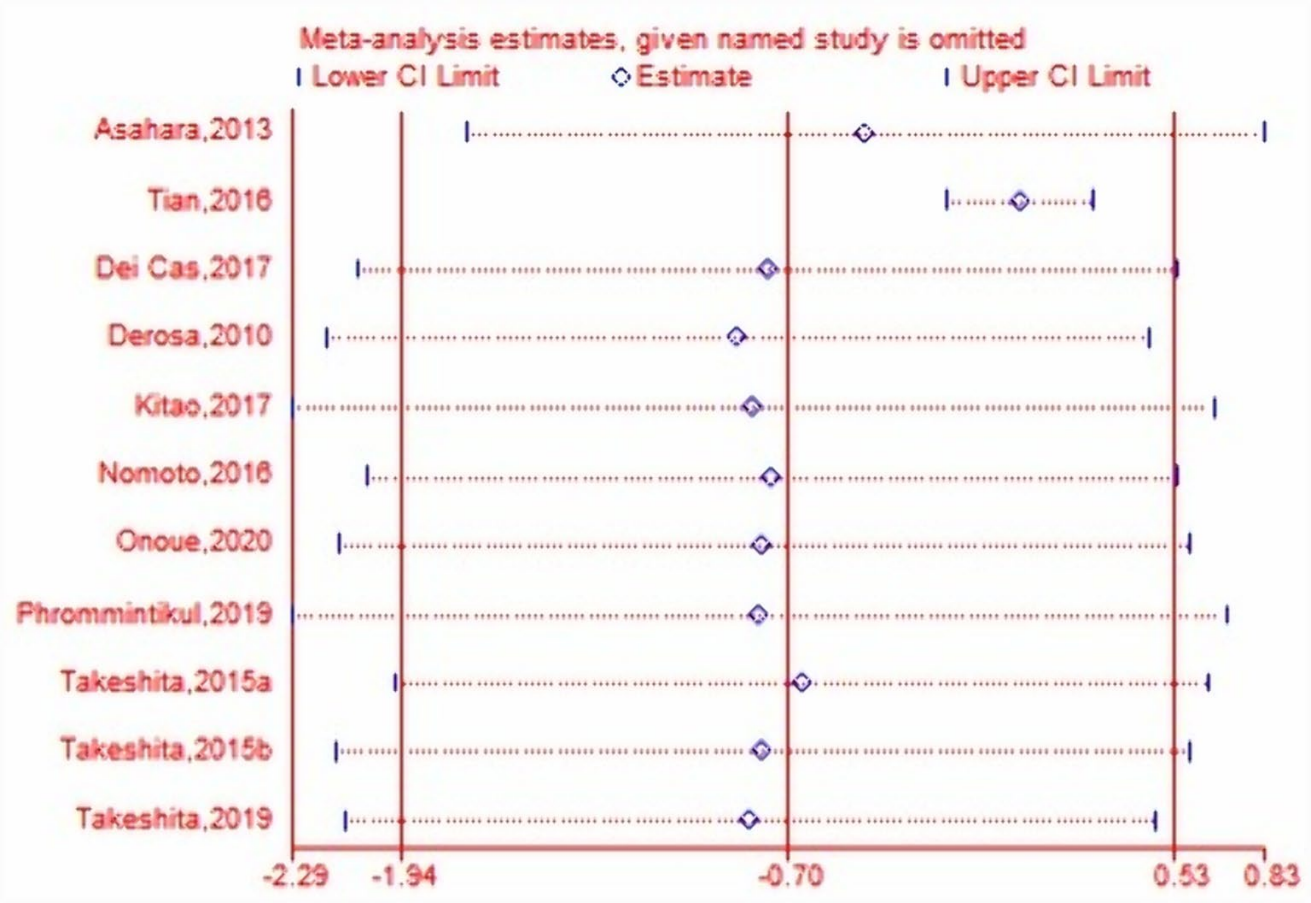
Leave-one-out sensitivity test for the effect of DPP-4i on TNF-alpha

**Fig. 5:**
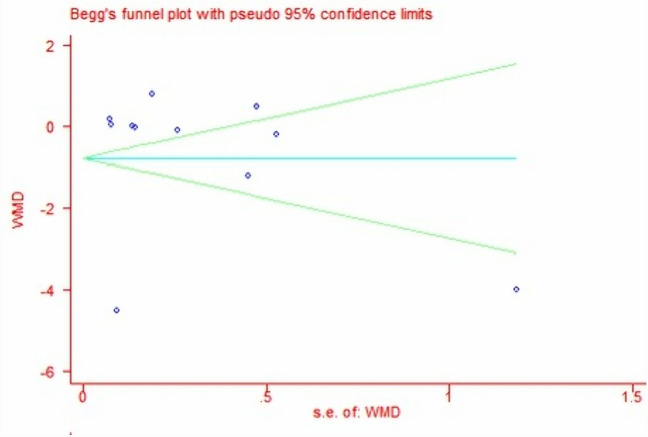
Assessment of publication bias in the studies for estimating the effect of DPP-4i on TNF-alpha

Meta-analysis was performed with sample size, mean change of TNF-alpha data, standard deviations (SD) between two intervention arms in each RCT. Pooled estimates suggested that DPP-4i did not significantly modulate TNF-alpha levels (WMD, − 0.70, 95% CI − 1.94 to 0.53, *P* = 0.26, *I*^2^ = 100%) in T2DM. DPP-4i produced a significant effect on TNF-alpha (WMD, − 4.50 pg/mL, 95% CI − 4.68 to − 4.32, *P* < 0.00001, *I*^2^ = 0%) when compared to placebo, while a comparable effect was induced (WMD, 0.10 pg/mL, 95% CI − 0.11 to 0.30, *P* = 0.35, *I*^2^ = 70.0%) when compared to other antidiabetic agents (Fig. 3). The pooled result proved to be stable after performing a sensitivity test (Fig. 4). In addition, no significant effect of DPP-4i was found on TNF-alpha after performing subgroup analysis on baseline HbA1c, age or lengths of follow-up (Table 3). Finally, no publication bias was observed after performing an Egger’s test (*P* = 0.413) and Begg’s test (Fig. 5) across 11 RCTs.

**Table 3:** Subgroup analysis on the correlation of DDP-4i with TNF-alpha in T2DM

Subgroups	Studies (*n*)	WMD, 95% CI	*I*^2^ (%)	*P*
HbA1c < 8.0%	6	− 0.03, − 0.32 to 0.26	59	0.85
HbA1c ≥ 8.0%	5	− 1.45, − 3.83 to 0.93	100	0.23
Age < 60	2	− 1.85, − 7.05 to 3.34	100	0.48
Age ≥ 60	9	− 0.03, − 0.25 to 0.18	68	0.75
Treatment duration ≤ 6 months	6	− 1.44, − 3.69 to 0.81	100	0.21
Treatment duration > 6 months	5	0.15, − 0.16 to 0.47	75	0.35

## Discussion

In the current meta-analysis, a comparison analysis on TNF-alpha levels between DPP-4i treatment with placebo or active comparators was performed in T2DM. The overall estimate showed that DPP-4i produced no significant effect on TNF-alpha levels in patients with T2DM. Administration of DPP-4i was associated with a significant reduction of TNF-alpha levels compared to placebo. This finding was consistent with a previous study, in which sitagliptin and vildagliptin produced an anti-inflammatory effect by reducing TNF-alpha concentrations in diabetic patients [12]. However, no further information on other comparisons was addressed on changes of TNF-alpha in T2DM. Considering the lack of comparative evidence on DPP-4i with other antidiabetic regimens, this study might add a detailed illustration of modulation on TNF-alpha levels.

Inflammatory response played a role underlying pathogenic mediator for an increased incidence of diabetic complications [13]. In addition, increased secretion of proinflammatory cytokines were commonly observed in patients with T2DM [14]. Among multiple cytokines, TNF-alpha served as a well-established inflammatory cytokine, mainly secreted by macrophages in adipose tissue. A low level of TNF-alpha activated defense system to prevent an invasion of infection. In contrast, an increased level of TNF-alpha contributed to the onset and development of inflammatory state [15]. Furthermore, TNF-alpha impaired insulin sensitivity through downregulating an expression of glucose transporter-4 [16]. Increased levels of TNF-alpha were also closely linked with the dysregulation of lipid metabolism, participating in the development of diabetic complications. Insulin resistance and lipid disorder could be improved when TNF-alpha level was reduced in diabetic setting [17, 18]. During the past years, several agents were approved to block an action of TNF-alpha for treatment of inflammatory diseases by the FDA. However, most of these agents resulted in a high level of triglyceride, and increased the incidence of diabetes and atherosclerotic diseases. Numerous efforts had been made to develop effective agents, and to explore underlying mechanisms to improve inflammation response in T2DM [19]. An increased understanding of the common pathology between diabetes and inflammation resulted in a focus on the therapeutic potential of antidiabetic drugs for treating inflammation in diabetic setting.

In fact, evidences from clinical trials suggested that antidiabetic treatment should not focus on modulating glucose levels alone. Additional benefits could be achieved if inflammatory response was alleviated during antidiabetic treatment was performed in T2DM [20]. Recently, novel agents of DPP-4i were introduced for treatment of T2DM, which presented additional benefits by targeting inflammatory signaling pathway [21]. In fact, DPP4 induced an inflammation response by activating mitogen-activated protein kinase (MAPK) signaling pathway [22]. In vitro experiment revealed that inflammation response could be alleviated by downregulating DPP4 expression. This also contributed to an improvement of insulin resistance [23]. In the current meta-analysis, evidence was provided that DPP-4i improved inflammation response by reducing TNF-alpha levels in T2DM. These pooled estimates were in line with a previous study in which saxagliptin favorably reduced TNF-alpha levels in diabetic condition [24].

The exact mechanism by which DPP-4i modulated TNF-alpha levels remained unclear in T2DM. Some studies had been performed to explore the underlying mechanisms. For example, teneligliptin could accelerate tubule regeneration and attenuated inflammatory response in models of acute kidney injury, which was mediated by preventing a breakdown of CXC chemokine ligand-12 [25]. Gemigliptin was also found to inhibit an expression of vascular adhesion molecules and TNF-alpha in human umbilical vein endothelial cells, selectively targeting AMPK signaling pathway [26]. In addition, saxagliptin alleviated an inflammation state by targeting the NOD-like receptor 3 (Nlrp3) pathway in diabetic setting [24]. Further study showed that an anti-inflammatory effect of DPP-4i did not seem to be restricted in conditions of T2DM. A stimulated inflammatory response was found in patients with heart failure and preserved ejection fraction (HFpEF), as characterized by an increase of inflammatory molecules including TNF-alpha. This contributed to a significant reduction of coronary endothelial function. Sitagliptin improved endothelial dysfunction by presenting an anti-inflammatory effect in HFpEF models [27]. Moreover, DPP-4i significantly alleviated inflammatory response in models of severe acute pancreatitis. This effect was partially mediated by inactivation of nuclear factor-kappa B (NF-κB) signaling pathway [28]. As TNF-alpha served as an important proinflammatory marker, it could be targeted especially in T2DM when developing new therapeutic agents. The motive for choosing DPP-4i over other available medications including either herbal might be based on the results from the recent study by Mokgalaboni et al. in 2024. In that study, no significant effect was demonstrated on TNF-alpha during treatment of T2DM [29].

The pooled estimate suggested that a reduction of TNF-alpha was not more pronounced in DPP-4i group than that of other active drugs group. The lack of significant difference might be explained by a composite action from multiple antidiabetic control arms. Active drugs including glimepiride, pioglitazone, dapagliflozin and liraglutide were compared in the control groups. These agents had been proved to present different effects in the modulation of inflammation response, thus affecting the pooled estimate of TNF-alpha changes. Traditional antidiabetic agents, including sulfonylureas and thiazolidinediones, might increase the levels of proinflammatory cytokines during treatment of T2DM [30]. In contrast, metformin, GLP-1 receptor agonists (GLP-1 RA) and sodium–glucose cotransporter 2 inhibitors (SGLT2i) significantly decreased the secretion of inflammatory factors including TNF-alpha [31]. A recent pooled estimate showed that GLP-1 RA and SGLT2i showed a stronger anti-inflammatory effect than other antidiabetic agents [32]. This potentially explained the non-significant effect of TNF-alpha when comparing DPP-4i with other active agents in T2DM [33].

## Strengths

Previously, our team found that DPP-4i had a favorable effect on adipose-specific adiponectin and C-reactive protein (CRP) in T2DM [34, 35]. In the current analysis, DPP-4i produced an anti-inflammatory effect by reducing TNF-alpha levels in diabetic patients from different regions. Furthermore, the effect on TNF-alpha was not affected by HbA1c, age or treatment duration during DPP-4i treatment. Given that chronic inflammation played an important role in the onset and progression of diabetes and related complications, the impact of DPP-4i on TNF-alpha might produce an additional benefit in diabetic patients [36]. It also provided an insight into the therapeutic implications in diabetes due to the potential protection from inflammation. Moreover, subgroup analysis was performed to explore the impact of baseline HbA1c, age and treatment duration. In addition, this study had potential significance on translational researches in diabetes field.

## Limitations

However, this meta-analysis had some limitations to be stated. Firstly, a small number of studies with a small sample of patients was a major limitation, which might result in an unstable estimate of treatment effects. Secondly, studies that used the combination of DPP-4i with other antidiabetic agents in the intervention group were not excluded due to the limited number of studies, which potentially produced an effect on TNF-alpha levels. However, participants in the control group (placebo or active drug) also received the same hypoglycemic drug as that of the DPP-4i group, which might have minimized the publication bias. Thirdly, the anti-inflammatory effect of different DPP-4i had not been discussed separately. In addition, the certainty of evidence assessed by GRADE system was rated very low, which also needed to be explored by involving more eligible RCTs with high quality. Finally, further trials were needed to elucidate the therapeutic value of DPP-4i in treating inflammatory diseases associated with diabetes.

## Conclusions

A significant effect of DPP-4i on TNF-alpha levels was present in T2DM when compared to placebo. DPP-4i produced no significant effect on TNF-alpha in comparison with active agents. Further studies with large samples should be performed to illustrate the impact of DPP-4i on TNF-alpha levels in T2DM.

### Supplementary Information

Supplementary Material 1.

Supplementary Material 2.

## Data Availability

All data generated or analyzed during this study are included in this published article and its supplementary information files.

## Abbreviations

CRP: C-reactive protein
DPP-4i: Dipeptidyl peptidase-4 inhibitors
GLP-1 RA: Glucagon-like peptide 1 receptor agonists
HFpEF: Heart failure with preserved ejection fraction
MAPK: Mitogen-activated protein kinase
Nlrp3: NOD-like receptor 3
NF-κB: Nuclear factor-kappaB
PRISMA: Preferred Reporting Items for Systematic reviews and meta-analysis
RCTs: Randomized controlled trials
SGLT2i: Sodium–glucose cotransporter 2 inhibitors
T2DM: Type 2 diabetes mellitus
TNF-alpha: Tumor necrosis factor alpha
