# Supplementary material for: Effect of dipeptidyl peptidase-4 inhibitors on tumor necrosis factor alpha levels in patients with type 2 diabetes mellitus
Source: Eur J Med Res. 2024 Jul 12;29:363. doi: 10.1186/s40001-024-01955-9 (PMC11241791; doi:10.1186/s40001-024-01955-9)
Supplement: Supplementary file 1 — Supplementary Material 1. [file 40001_2024_1955_MOESM1_ESM.docx]

**PubMed**

| ID | Query | Results |
| --- | --- | --- |
| #1 | ((((((((randomized controlled trial) OR (Controlled Clinical Trials)) OR (random allocation)) OR (double-blind)) OR (single-blind)) OR (placebo)) OR (randomly)) OR (randomized)) OR (RCT) | 1948033 |
| #2 | ((((((((((((((((((((((((Dipeptidyl Peptidase IV Inhibitors) OR (Dipeptidyl-Peptidase IV Inhibitors)) OR (DPP-4 Inhibitor)) OR (DPP 4 Inhibitor)) OR (Inhibitor, DPP-4)) OR (DPP-IV Inhibitor)) OR (DPP IV Inhibitor)) OR (Inhibitor, DPP-IV)) OR (DPP-4 Inhibitors)) OR (DPP 4 Inhibitors)) OR (DPP-IV Inhibitors)) OR (DPP IV Inhibitors)) OR (DPP4 Inhibitors)) OR (Dipeptidyl Peptidase 4 Inhibitor)) OR (Dipeptidyl-Peptidase IV Inhibitor)) OR (Dipeptidyl Peptidase IV Inhibitor)) OR (Inhibitor, Dipeptidyl-Peptidase IV)) OR (Dipeptidyl-Peptidase 4 Inhibitor)) OR (Inhibitor, Dipeptidyl-Peptidase 4)) OR (Dipeptidyl-Peptidase 4 Inhibitors)) OR (Dipeptidyl Peptidase 4 Inhibitors)) OR (Gliptins)) OR (DPP4 Inhibitor)) OR (Inhibitor, DPP4)) OR (Gliptin) | 11002 |
| #3 | (((((((((((((((((((((((((((((((Diabetes Mellitus, Type 2) OR (Diabetes Mellitus, Adult-Onset)) OR (Adult-Onset Diabetes Mellitus)) OR (Diabetes Mellitus, Adult Onset)) OR (Diabetes Mellitus, Ketosis-Resistant)) OR (Diabetes Mellitus, Ketosis Resistant)) OR (Ketosis-Resistant Diabetes Mellitus)) OR (Diabetes Mellitus, Non Insulin Dependent)) OR (Diabetes Mellitus, Non-Insulin-Dependent)) OR (Non-Insulin-Dependent Diabetes Mellitus)) OR (Diabetes Mellitus, Stable)) OR (Stable Diabetes Mellitus)) OR (Diabetes Mellitus, Type II)) OR (NIDDM)) OR (Diabetes Mellitus, Noninsulin Dependent)) OR (Diabetes Mellitus, Maturity-Onset)) OR (Diabetes Mellitus, Maturity Onset)) OR (Maturity-Onset Diabetes Mellitus)) OR (Maturity Onset Diabetes Mellitus)) OR (MODY)) OR (Diabetes Mellitus, Slow-Onset)) OR (Diabetes Mellitus, Slow Onset)) OR (Slow-Onset Diabetes Mellitus)) OR (Type 2 Diabetes Mellitus)) OR (Noninsulin-Dependent Diabetes Mellitus)) OR (Noninsulin Dependent Diabetes Mellitus)) OR (Maturity-Onset Diabetes)) OR (Diabetes, Maturity-Onset)) OR (Maturity Onset Diabetes)) OR (Type 2 Diabetes)) OR (Diabetes, Type 2)) OR (Diabetes Mellitus, Noninsulin-Dependent) | 279577 |
| #4 | #1 AND #2 AND #3 | 2288 |

**Embase**

| ID | Query | Results |
| --- | --- | --- |
| #1 | 'crossover procedure':de OR 'double-blind procedure':de OR 'randomized controlled trial':de OR 'single-blind procedure':de OR random*:de,ab,ti OR factorial*:de,ab,ti OR crossover*:de,ab,ti OR ((cross NEXT/1 over*):de,ab,ti) OR placebo*:de,ab,ti OR ((doubl* NEAR/1 blind*):de,ab,ti) OR ((singl* NEAR/1 blind*):de,ab,ti) OR assign*:de,ab,ti OR allocat*:de,ab,ti OR volunteer*:de,ab,ti | 3338570 |
| #2 | rat:ti OR rats:ti OR mouse:ti OR mice:ti OR swine:ti OR porcine:ti OR murine:ti OR sheep:ti OR lambs:ti OR pigs:ti OR piglets:ti OR rabbit:ti OR rabbits:ti OR cat:ti OR cats:ti OR dog:ti OR dogs:ti OR cattle:ti OR bovine:ti OR monkey:ti OR monkeys:ti OR trout:ti OR marmoset:ti | 2620157 |
| #3 | #1 NOT #2 | 3166062 |
| #4 | ‘Dipeptidyl Peptidase IV Inhibitors’ OR 'Dipeptidyl-Peptidase IV Inhibitors' OR 'DPP-4 Inhibitor' OR 'DPP 4 Inhibitor' OR 'Inhibitor, DPP-4' OR 'DPP-IV Inhibitor' OR 'DPP IV Inhibitor' OR 'Inhibitor, DPP-IV' OR 'DPP-4 Inhibitors' OR 'DPP 4 Inhibitors' OR 'DPP-IV Inhibitors' OR 'DPP IV Inhibitors' OR 'DPP4 Inhibitors' OR 'Dipeptidyl Peptidase 4 Inhibitor' OR 'Dipeptidyl-Peptidase IV Inhibitor' OR 'Dipeptidyl Peptidase IV Inhibitor' OR 'Inhibitor, Dipeptidyl-Peptidase IV' OR 'Dipeptidyl-Peptidase 4 Inhibitor' OR 'Inhibitor, Dipeptidyl-Peptidase 4' OR 'Dipeptidyl-Peptidase 4 Inhibitors' OR 'Dipeptidyl Peptidase 4 Inhibitors' OR 'Gliptins' OR 'DPP4 Inhibitor' OR 'Inhibitor, DPP4' OR 'Gliptin' | 20021 |
| #5 | 'Diabetes Mellitus, Type 2' OR 'Diabetes Mellitus, Adult-Onset' OR 'Adult-Onset Diabetes Mellitus' OR 'Diabetes Mellitus, Adult Onset' OR 'Diabetes Mellitus, Ketosis-Resistant' OR 'Diabetes Mellitus, Ketosis Resistant' OR 'Ketosis-Resistant Diabetes Mellitus' OR 'Diabetes Mellitus, Non Insulin Dependent' OR 'Diabetes Mellitus, Non-Insulin-Dependent' OR 'Non-Insulin-Dependent Diabetes Mellitus' OR 'Diabetes Mellitus, Stable' OR 'Stable Diabetes Mellitus' OR 'Diabetes Mellitus, Type II' OR 'NIDDM' OR 'Diabetes Mellitus, Noninsulin Dependent' OR 'Diabetes Mellitus, Maturity-Onset' OR 'Diabetes Mellitus, Maturity Onset' OR 'Maturity-Onset Diabetes Mellitus' OR 'Maturity Onset Diabetes Mellitus' OR 'MODY' OR 'Diabetes Mellitus, Slow-Onset' OR 'Diabetes Mellitus, Slow Onset' OR 'Slow-Onset Diabetes Mellitus' OR 'Type 2 Diabetes Mellitus' OR 'Noninsulin-Dependent Diabetes Mellitus' OR 'Noninsulin Dependent Diabetes Mellitus' OR 'Maturity-Onset Diabetes' OR 'Diabetes, Maturity-Onset' OR 'Maturity Onset Diabetes' OR 'Type 2 Diabetes' OR 'Diabetes, Type 2' OR 'Diabetes Mellitus, Noninsulin-Dependent' | 417077 |
| #6 | #3 AND #4 AND #5 | 4812 |

**Cochrane library**

| ID | Query | Results |
| --- | --- | --- |
| #1 | ((((((((randomized controlled trial) OR (Controlled Clinical Trials)) OR (random allocation)) OR (double-blind)) OR (single-blind)) OR (placebo)) OR (randomly)) OR (randomized)) OR (RCT) | 1780779 |
| #2 | ((((((((((((((((((((((((Dipeptidyl Peptidase IV Inhibitors) OR (Dipeptidyl-Peptidase IV Inhibitors)) OR (DPP-4 Inhibitor)) OR (DPP 4 Inhibitor)) OR (Inhibitor, DPP-4)) OR (DPP-IV Inhibitor)) OR (DPP IV Inhibitor)) OR (Inhibitor, DPP-IV)) OR (DPP-4 Inhibitors)) OR (DPP 4 Inhibitors)) OR (DPP-IV Inhibitors)) OR (DPP IV Inhibitors)) OR (DPP4 Inhibitors)) OR (Dipeptidyl Peptidase 4 Inhibitor)) OR (Dipeptidyl-Peptidase IV Inhibitor)) OR (Dipeptidyl Peptidase IV Inhibitor)) OR (Inhibitor, Dipeptidyl-Peptidase IV)) OR (Dipeptidyl-Peptidase 4 Inhibitor)) OR (Inhibitor, Dipeptidyl-Peptidase 4)) OR (Dipeptidyl-Peptidase 4 Inhibitors)) OR (Dipeptidyl Peptidase 4 Inhibitors)) OR (Gliptins)) OR (DPP4 Inhibitor)) OR (Inhibitor, DPP4)) OR (Gliptin) | 2710 |
| #3 | (((((((((((((((((((((((((((((((Diabetes Mellitus, Type 2) OR (Diabetes Mellitus, Adult-Onset)) OR (Adult-Onset Diabetes Mellitus)) OR (Diabetes Mellitus, Adult Onset)) OR (Diabetes Mellitus, Ketosis-Resistant)) OR (Diabetes Mellitus, Ketosis Resistant)) OR (Ketosis-Resistant Diabetes Mellitus)) OR (Diabetes Mellitus, Non Insulin Dependent)) OR (Diabetes Mellitus, Non-Insulin-Dependent)) OR (Non-Insulin-Dependent Diabetes Mellitus)) OR (Diabetes Mellitus, Stable)) OR (Stable Diabetes Mellitus)) OR (Diabetes Mellitus, Type II)) OR (NIDDM)) OR (Diabetes Mellitus, Noninsulin Dependent)) OR (Diabetes Mellitus, Maturity-Onset)) OR (Diabetes Mellitus, Maturity Onset)) OR (Maturity-Onset Diabetes Mellitus)) OR (Maturity Onset Diabetes Mellitus)) OR (MODY)) OR (Diabetes Mellitus, Slow-Onset)) OR (Diabetes Mellitus, Slow Onset)) OR (Slow-Onset Diabetes Mellitus)) OR (Type 2 Diabetes Mellitus)) OR (Noninsulin-Dependent Diabetes Mellitus)) OR (Noninsulin Dependent Diabetes Mellitus)) OR (Maturity-Onset Diabetes)) OR (Diabetes, Maturity-Onset)) OR (Maturity Onset Diabetes)) OR (Type 2 Diabetes)) OR (Diabetes, Type 2)) OR (Diabetes Mellitus, Noninsulin-Dependent) | 97612 |
| #4 | #1 AND #2 AND #3 | 2299 |
